# Pathophysiology of Congenital Anomalies of the Kidney and Urinary Tract: A Comprehensive Review

**DOI:** 10.3390/cells13221866

**Published:** 2024-11-11

**Authors:** Maximilian Brockwell, Sean Hergenrother, Matthew Satariano, Raghav Shah, Rupesh Raina

**Affiliations:** 1 Department of Medicine, Northeast Ohio Medical University, Rootstown, OH 44272, USA; mbrockwell@neomed.edu (M.B.); shergenrother@neomed.edu (S.H.); msatariano@neomed.edu (M.S.); rshah10@neomed.edu (R.S.); 2 Akron Nephrology Associates, Cleveland Clinic Akron General Medical Center, Akron, OH 44307, USA; 3 Department of Nephrology, Akron Children’s Hospital, Akron, OH 44308, USA

**Keywords:** congenital anomalies of the kidney and urinary tract, CAKUT, renal, kidney, dysplasia, vesicoureteral reflux, bladder–exstrophy–epispadias complex, horseshoe kidney, obstructive uropathy

## Abstract

Congenital anomalies of the kidney and urinary tract (CAKUT) represent a broad range of diseases with differing mechanisms, clinical presentations, and prognoses. With an estimated prevalence of between 4 and 60 per 10,000 births, CAKUT represents a sizable number of patients for pediatric and adult nephrologists as therapies have progressed, allowing longer life spans. Many CAKUT disorders are associated with genetic mutations, and with advances in genomic sequencing, these genes are being identified at an increasing rate. Understanding these mutations provides insight into these conditions’ molecular mechanisms and pathophysiology. In this article, we discuss the epidemiology, presentation, and outcomes of CAKUT in addition to our current understanding of genetic and molecular mechanisms in these diseases.

## 1. Introduction

Congenital anomalies of the kidney and urinary tract (CAKUT) encompass a variety of developmental disorders stemming from malformations of the kidneys and their outflow tracts. These anomalies represent a significant healthcare burden affecting between 4 and 60 per 10,000 live births, which equates to 20% of end-stage renal disease (ESRD) in adults [1,2]. CAKUT disorders include nephropathies such as renal aplasia, hypoplasia, dysplasia, oligomeganephronia, ectopic kidneys, and horseshoe kidney. Uropathies such as multiple ureters, vesicoureteral reflux, bladder–exstrophy–epispadias complex, posterior urethral valve, and ureteroceles are also described in CAKUT. The primary breakthroughs in patient survival have mainly been advancements in urologic surgery, dialysis, and transplants. With an increased lifespan, CAKUT has transitioned from a primarily pediatric condition to a condition that is becoming more prevalent in adults. Despite recognition of these disorders, genetic testing has lagged in consistently providing robust prognostic and treatment guidance. Although some genetic causes remain unknown, a deeper understanding of genetic and environmental risk factors is essential to provide diagnostic insight into management and treatment. This review explores our current understanding of the pathophysiology of CAKUT and highlights recent research into genetic etiologies.

## 2. Background

To understand the pathophysiology of CAKUT, it is essential to first grasp the normal progression of renal development. The kidney has three separate iterations: the pronephros, mesonephros, and metanephros, with each stage building upon the function and coordination of the prior. Renal development comprises two systems, the proximal and distal collecting systems. In the metanephros, these join to become the nephron and its outflow tract through the renal pelvis, ureters, bladder, and urethra. The proximal collecting system encompasses the first section of the nephron from the glomerulus through the distal tubules, and the distal collecting system comprises the collecting ducts. These systems interact at each embryonic and fetal development step and become highly organized. This complex system allows for precise temporal and spatial control, allowing the kidneys to ultimately ascend and take their final place in the rostral retroperitoneal space.

**Figure 1:**
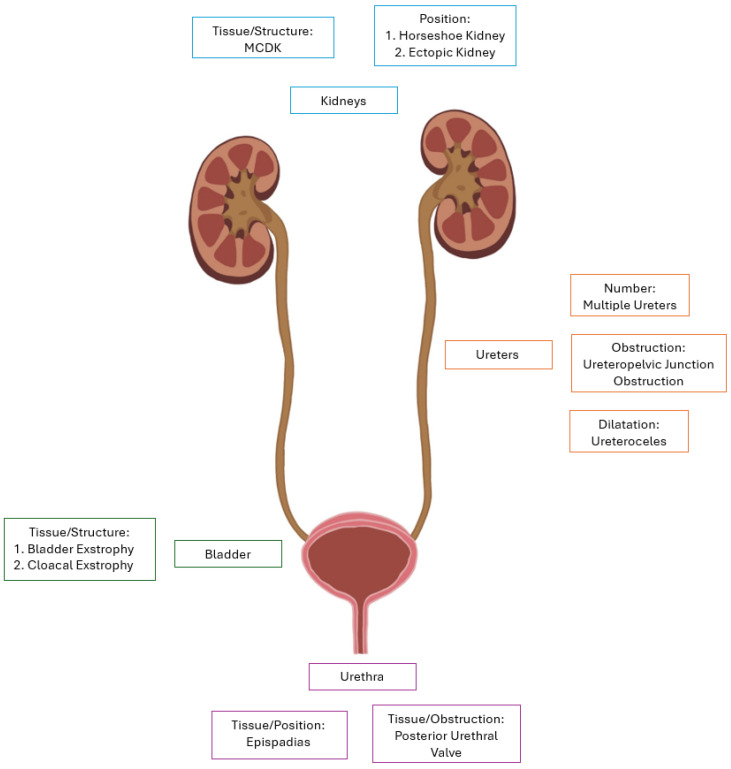
Visualization of various CAKUT pathologies in relation to the effected structures. Graphic created by Lia Hergenrother.

Renal development begins with the pronephros, which arises from the intermediate mesoderm in week three of development [3]. The initial tubules, known as nephrotomes, form and coalesce into pronephric ducts. However, these structures are short-lived as the pronephric ducts induce the formation of the mesonephric tubules. By the end of the fourth week of gestation the pronephric ducts have completely regressed, and the mesonephric duct has formed along with the mesonephros caudally. The mesonephric (Wolffian) duct plays a crucial role in kidney development, and also forms the vas deferens in the male reproductive tract. The inferior aspect of the mesonephric duct gives rise to the ureteric bud, which invades the surrounding metanephric mesenchyme in response to induction signals. From here, the ureteric bud will undergo subsequent branching and segmentation to form the mature collecting duct system inside the metanephros and eventually induce the metanephros to become the mature kidney [4]. The lower collecting system consists of collecting tubules, minor and major calyces, the renal pelvis, and ureters. Once the upper and lower collecting systems are joined around week 16, the fetus begins to make urine, which is crucial for maintaining appropriate amounts of amniotic fluid and promoting proper fetal lung development. Each step in development is tightly controlled by transcription factors and any errors in expression can lead to severe consequences in these organ systems (Figure 1).

The diseases discussed in this review fall into two categories: nephropathies and uropathies. Nephropathies arise from a defect during renal development, whereas uropathies arise in structures distal to the kidneys (Table 1). The pathophysiology, clinical presentations, and genetic causes of CAKUT vary significantly, and are summarized in Table 2. Of note, ciliopathies, such as autosomal polycystic kidney disease, by definition, are not classified as CAKUT and thus are not discussed.

## 3. CAKUT Diagnoses

### 3.1. Nephropathies

#### 3.1.1. Aplasia and Dysplasia

##### Epidemiology

Renal agenesis refers to the absence of either one or both kidneys at birth. Unilateral renal agenesis is often asymptomatic and more common, with an incidence of 1 per 1000 births. Bilateral agenesis is incompatible with life and rarer, with an incidence of 1–3 per 10,000 births [5]. Multicystic dysplastic kidney (MCDK) carries an incidence of 1 per 4300 births with a slight male predominance [6].

##### Clinical Presentation

Unilateral renal agenesis is often benign and discovered incidentally, although it is occasionally associated with other genetic syndromes. In Zinner Syndrome, a usually asymptomatic disorder exclusive to males, dysfunction in the mesonephric duct leads to unilateral renal agenesis, ipsilateral seminal vesicle cysts, and ipsilateral ejaculatory duct obstruction [7]. In females, an anatomic defect in the mesonephric duct leads to Herlyn-Werner–Wunderlich syndrome, or obstructed hemivagina and ipsilateral renal anomaly (OHVIRA). This syndrome presents with a triad of uterine malformation, ipsilateral blind or atretic hemivagina, and renal agenesis/hypoplasia [8]. Complications of OHVIRA include Gartner duct pseudocysts with communicating uteri (Herlyn–Werner syndrome), obstructed/blind hemivagina with hematocolpos (Wunderlich syndrome), uterine didelphys, or unilateral cervicovaginal atresia [9]. Mullerian aplasia, also known as Mayer-Rokitansky–Kuster–Hauser syndrome (MRKHS), is defined as the absence of the uterus, cervix, and upper vagina in females. MRKHS is associated with renal malformations, including aplasia, in ~30% of cases [10].

Bilateral renal agenesis occurs less frequently than unilateral and is rapidly fatal due to the absence of amniotic fluid production leading to the Potter sequence. Pulmonary hypoplasia is a serious complication, and early infant demise is common.

Multicystic dysplastic kidney is characterized by numerous cystic lesions compromising renal function caused by failed induction of the metanephric mesenchyme. In the case of bilateral MCDK, Potter sequence and fetal demise are likely. Unilateral MCDK may prompt further genetic testing for concomitant conditions, including Zellweger, VACTERL, Eagle–Barret syndrome, branchio-oto-renal syndrome, renal coloboma, and renal–hepatic–pancreatic dysplasia [11].

##### Pathophysiology

Unilateral renal agenesis results from a failure of the ureteric bud to form. Studies determining signal abnormalities in the distal mesonephric duct and its derivatives have yielded some genetic explanations. Research into genes associated with Zinner syndrome is limited; however, variants of CFTR and ADGRG2 were identified in men with X-linked azoospermia secondary to congenital absence of vas deferens, and renal agenesis is associated with congenital unilateral absence of the vas deferens [12,13]. In females, the mesonephric duct induces the development of the paramesonephric duct, and if disrupted, can lead to abnormalities in the urogenital organs. For instance, in OHVIRA the CHD1L, TRIM32, RET, and WNT4 genes may be associated with renal agenesis [14]. In MRKHS, WNT4 was historically hypothesized as the primary driver of disease. However, recent studies have shown that the PAX8, BMP4, BMP7, TBX6, HOXA10, EMX2, and WNT9B genes may play a role [15]. Further research is needed to analyze the interplay of these genes and their products. Another gene of interest is WT1 [16,17]. While originally identified in the pathophysiology of Denys–Drash syndrome and Frasier syndrome [18], a missense WT1 mutation has also been observed in the agenesis of fetal kidneys [16].

Bilateral renal agenesis has been associated with multiple gene mutations, including heterozygous mutations in ITGA8 (renal dysplasia 1), FGF20 (renal dysplasia 2), GREB1L (renal dysplasia 3), and DSTYK (CAKUT1) [9]. Other genes that have been identified in bilateral renal agenesis include ANOS1, EYA1, and RET [6]. ANOS1 is expressed during renal development and is also associated with hypogonadotropic hypogonadism (Kallmann syndrome) [19]. EYA1 is associated with branchio-oto-renal syndrome, which can cause numerous renal deficits, including agenesis, hypoplasia, dysplasia, oligomeganephronic hypoplasia, VUR, and UPJ obstruction [20]. In renal embryology, RET is normally activated in the developing Wolffian duct through WNT11 production of its ligand GDNF [21,22,23]. Paradoxically, mutations causing both activation and inactivation of the RET proto-oncogene have been linked to renal aplasia, emphasizing the importance of the precise control of RET in kidney development [6,21].

MCDK pathophysiology has been linked to dysregulation of PAX2 and TCF2 as well as other potential genes [11]. The calcineurin-NFAT signaling cascade, expressed in the ureteric bud, is crucial for normal function and development of urogenital organs, which, when dysregulated, leads to the abnormalities observed in MCDK [24,25].

#### 3.1.2. Hypoplasia and Oligomeganephronia

##### Epidemiology

Renal hypoplasia is characterized by decreased number and volume of kidney lobes, resulting in diminished surface area for filtration, collection, and excretion. These disorders are categorized into three groups: simple hypoplasia, oligomeganephronic hypoplasia (oligomeganephronia), and segmental hypoplasia [26]. Estimates on the prevalence of simple hypoplasia are uncertain due to clinically asymptomatic course. However, oligomeganephronia and segmental hypoplasia are rare, with the latter being more common in women (72% of cases) [26,27].

##### Clinical Presentation

Simple renal hypoplasia is defined as a kidney less than 50% of expected weight with preserved architecture, although a definition of 5 or fewer calyces may be more appropriate. Despite reduction in size, a single hypoplastic kidney usually exhibits normal function, although this may lead to an increased risk of hypertension. In contrast, bilateral simple hypoplastic kidneys may increase the risk of progression to early kidney failure [26].

Oligomeganephronic hypoplasia is characterized by bilateral renal hypoplasia with a low number of enlarged nephrons [27]. Oligomeganephronia has a bimodal distribution in age of presentation, with one study demonstrating 58% of patients diagnosed before the age of 1, and the remaining 42% remaining asymptomatic until 10–15 years old [26,28]. The condition frequently progresses to proteinuria, hyperfiltration, and eventual end-stage renal disease, notably in the absence of hypertension [29]. While the mechanism is unknown, oligomeganephronic hypoplasia is associated with several rare congenital disorders such as renal–coloboma syndrome, branchio-oto-renal syndrome (BOR), Townes–Brocks syndrome, acrorenal syndrome, and Seckel syndrome [26].

Segmental hypoplasia is described as either unilateral or bilateral renal hypoplasia, most frequently in pediatric females, severe renin-mediated hypertension, with or without urinary tract infection (UTI). Segmental hypoplasia is distinguished from other hypoplastic presentations by hypoplastic blood vessels, widely spaced collecting ducts, glomerular sclerosis, and tubular atrophy on histologic exam [26,30].

##### Pathophysiology

Multiple genes have been implicated in simple renal hypoplasia, including BMP2, BMP4, and the Alk3 receptor gene. In mouse models, downregulation of Alk3 resulted in a simple renal hypoplasia phenotype via a downstream decrease in Osr1 and SIX2, which are expressed in the metanephric mesenchyme at sites of nephrogenesis [31,32]. BMP signaling regulates nephron number and is likely involved in all hypoplastic phenotypes [33,34].

Although the mechanism is uncertain, oligomeganephronic hypoplasia is hypothesized to be related to congenital causes or pyelonephritis. Oligomeganephronic hypoplasia is seen in several rare congenital disorders associated with mutations in PAX2, EAY1, SALL1, RET1, and chromosome 4 deletions [26]. For instance, in renal coloboma syndrome, also known as papillorenal syndrome, 50% of cases are associated with PAX2 mutations. This pediatric syndrome involves optic nerve dysplasia (coloboma) as well as renal hypoplasia, multicystic dysplastic kidney, or oligomeganephronia and is usually fatal [35]. EAY1 mutations are associated with branchio-oto-renal syndrome, as described in prior discussions on renal agenesis [20]. SALL1 mutations have been identified in Townes–Brocks syndrome, which is characterized by renal, ear, anal, and limb malformations [36]. Mutations in the RET gene may interfere with the branching of the ureteric bud, leading to a decreased number of nephrons [21], and animal studies found RET knockout mice were anephric [37]. Furthermore, RET1 mutations may contribute to acrorenal syndrome which is characterized by distal limb malformations in addition to CAKUT [26]. Chromosome 4 abnormalities contributing to Seckel syndrome were also associated with oligomeganephronia and horseshoe kidney [38,39,40].

In contrast to other categories of renal hypoplasia, segmental hypoplasia appears to be acquired based on its adult onset. There is limited data on specific mutations with unclear underlying genetic etiologies and the likelihood of environmental factors playing a role [41,42].

#### 3.1.3. Positional Anomalies of the Kidneys (Horseshoe Kidney, Ectopic Kidney, Pancake Kidney, Malrotation)

##### Epidemiology

Positional anomalies of the kidney occur when the kidneys are not positioned within the rostral retroperitoneum. Horseshoe kidneys (HSK) have an incidence of 1 in 400, making them the most common renal fusion defect [43]. Ectopic kidneys are less common, occurring at a rate of 1 in 1000 births, although reported incidence varies widely. Malrotation is rare, and epidemiologic data is limited [44].

##### Clinical Presentation

Many cases of abnormally positioned kidneys are asymptomatic and are often discovered incidentally on imaging. In HSK, renal function is generally conserved, though the function may be slightly diminished in an ectopic kidney. If the patient does experience symptoms, the most common presentations include recurrent urinary tract infections, abdominal pain, vesicoureteric reflux, and nephrolithiasis [43,44]. There is also an increased risk of malignancy such as Wilms tumor in horseshoe kidneys [45].

Ectopic kidneys usually present with a single normally-located kidney, with the other kidney located in an aberrant position. The majority of ectopic kidneys are located in the pelvis, though rarely they occur in the thoracic cavity and in this case are generally associated with a diaphragmatic hernia [44,46].

Malrotated kidneys are often asymptomatic; however, complications secondary to abnormal positioning of the kidney and ureters may occur, including UTI, ureteropelvic obstruction, and hydronephrosis. Anterior positioning of the renal pelvis is the most common presentation; however, lateral positioning due to aberrant rotation is also observed [47,48].

Pancake kidney is a very rare congenital anomaly in which both kidneys fuse into one mass, usually situated in the pelvis. L-shaped kidneys represent another example of ectopic fusion, which is characterized by one kidney crossing the midline during development, thereby forming an “L-shape” fused kidney. In both these fusion anomalies of the kidney, patients are generally asymptomatic but may experience UTIs, nephrolithiasis, or obstructive symptoms due to abnormal positioning and ureteral kinking [49,50,51].

##### Pathophysiology

During normal development, the kidneys ascend between weeks 6 and 9 gestation to their final position between T12 and T3 in the abdomen. However, in HSK, the inferior renal poles remain fused, causing the kidney to become trapped below the inferior mesenteric artery at L3 [43]. The mechanism leading to HSK is uncertain; however, it is theorized that it may result from abnormal signaling during metanephros formation between the ureteric bud and the metanephric mesenchymal blastema [52]. Another theory suggests that migration of metanephric tissue during development across the midline leads to fusion [53]. HSKs are associated with certain syndromic conditions, such as Edward, Turner, and Down syndrome [43,54]. Environmental toxins such as Thalidomide may contribute to horseshoe kidneys and are also associated with ectopic kidneys [52].

Genetic mutations related to ectopic kidneys are not well established. However, ectopic kidneys are associated with other genitourinary abnormalities such as vaginal atresia, imperforate hymen, hypoplastic uteri, and rudimentary fallopian tubes/ovaries [55,56].

### 3.2. Uropathies

#### 3.2.1. Multiple Ureters and Vesicoureteral Reflux

##### Epidemiology

A duplex collecting system is the most common congenital abnormality of the urinary tract, impacting anywhere from 1–5% of the population. There is a female predominance, with some studies reporting a 2:1 ratio [57]. Notably, VUR is present in nearly 56% of patients with a completely duplicated renal system and is found in the lower ureter nearly 75% of the time [58].

##### Clinical Presentation

Multiple ureters represent a broad spectrum of phenotypes, from asymptomatic to severe obstruction and hydronephrosis. An estimated 40% of patients with duplex kidneys are symptomatic; however, this number may be overestimated due to the under-identification of asymptomatic cases [59]. If symptomatic, common manifestations of multiple ureters include vesicoureteral reflux (VUR), hydronephrosis, ureteroceles, calculi, or yo-yo reflux [60].

VUR is a consequence of abnormal development of the ureterovesical junction. The ureter normally enters the bladder at an oblique angle, preventing the backward flow of urine. In cases of VUR, a shortened intravesical ureteral tunnel leads to malfunction of this mechanism, permitting the retrograde flow of urine from the bladder to the ureters and kidneys, increasing the risk of infection and damage to the kidneys [61].

##### Pathophysiology

Multiple ureters are thought to arise after duplication of the ureteric bud. There are many ways in which the ureteric bud may duplicate, and as a result, many genetic mechanisms are implicated. At the molecular level, multiple ureters can form as a result of abnormal interactions between the metanephric mesenchyme and ureteric bud, causing incorrect timing of branching or infiltration of a second ureteric bud. Numerous genes have been implicated in duplex ureter formation, including PAX2, SALL1, RET, EYA1, GDNF, SLIT/ROBO, and GATA3 [60].

In addition to RET’s role in nephron branching, increased expression of RET’s ligand Gdnf in the rostral nephrogenic cord at the time of ureter induction is associated with ureteric bud defects [60]. Another ligand and receptor pair, SLIT/ROBO, are known in the axonal repulsion pathway [62]. Mutations in the receptor ROBO2 contribute to the pathogenesis of VUR and multiple ureters through loss of pathfinding of the ureteric bud and through dysregulation of Gdnf [60]. Therefore, it may be extrapolated that the interactions between SLIT2/ROBO2 and Gdnf, along with their regulators, Foxc1, Foxc2, and Sox11, could contribute to the pathogenesis of multiple ureters. Separately, PAX2 targets GATA3 to transcriptionally activate RET and beta-catenin [60]. RET is also negatively regulated by BMP4, which is suppressed by GREM1 activity. Therefore, both under-expression of BMP4 and over-expression of GREM1 can contribute to duplex kidney formation due to downstream effects on RET signaling [60].

The transcriptional regulator Beta-catenin, which is also involved in cell-cell adhesion, may play a role in the formation of duplex ureters. Beta-catenin inactivation has been linked to decreased nephron branching through its downstream effects on Emx2, Lim1, and c-ret [63]. Additionally, the angiotensin type 2 receptor gene (agtr2), responsible for preventing aberrant development of ureteral buds, has been observed to lead to duplex collecting systems when mutated in mouse models [64].

Other gene mutations associated with vesicoureteral reflux (VUR) include ROBO2, PAX2, AGTR2, RET, and TNXB, which are involved in the development of the urinary tract and connective tissues as discussed above [61,62,65].

#### 3.2.2. Bladder-Exstrophy-Epispadias-Complex

##### Epidemiology

Bladder–exstrophy–epispadias complex (BEEC) represents a spectrum of congenital defects of the lower urinary tract and surrounding structures, including the abdominal wall, pelvis, genitalia, anus, and spine [66,67,68]. These conditions are rare, with epispadias, bladder exstrophy, and cloacal exstrophy occurring in 2, 4, and 0.5–1 births per 100,000, respectively. Epispadias and bladder exstrophy occur more often in males; however, cloacal exstrophy is more common in females [66,68].

##### Clinical Presentation

Epispadias is the least severe manifestation of BEEC, presenting with failed closure of the urethra. This results in a more proximal urethral meatus on the dorsal penis in males or more proximal to the clitoris in females. These disorders are often managed surgically early in life, with long-term complications including incontinence [67,68].

Bladder exstrophy occurs when the interior surface of the bladder plate is exposed through the ventral abdominal wall and is generally accompanied by epispadias. Similarly to epispadias, incontinence is a frequent long-term complication, and multiple procedures are often required to improve urinary control. Pyelonephritis is common in these patients, likely due to VUR from increased post-surgical resistance at the bladder outlet. Additionally, these patients are more susceptible to the development of hydronephrosis, renal scarring, chronic kidney disease (CKD), and eventual renal failure [66,67,68]. However, more recent data has indicated that long-term renal function may not be as impaired as previously believed [69].

Cloacal exstrophy is the most severe presentation of BEEC, with an abdominal wall defect exposing both bladder and bowel, with additional pelvic bone and genital anomalies. Numerous defects are associated with cloacal exstrophy, including omphalocele, vertebral defects, imperforate anus, intestinal malrotation or duplication, in addition to other CAKUT diseases such as renal agenesis, ectopic kidney, hydronephrosis, and genital anomalies [68,70].

##### Pathophysiology

The precise cause of BEEC malformations has not been determined; however, there are multiple theories that explain these congenital anomalies. One theory suggests cloacal exstrophy may be caused by failed migration of the mesenchyme due to overdevelopment of the cloacal membrane. Without support from the mesenchyme, the cloacal membrane is prone to rupture. If this occurs prior to the formation of the urorectal septum, cloacal exstrophy results in the herniation of bowel and bladder through the abdominal wall. However, if this rupture occurs after the abdominal mesenchyme has migrated medially prior to mesenchymal migration in the urethra, epispadias may occur [49,51]. An alternative theory suggests that a defect in the formation of the pelvic ring allows exstrophy of these organs [71].

There appears to be some hereditary basis of BEEC based on familial and twin studies; however, the exact mode of inheritance is uncertain [67]. Multiple chromosomal abnormalities have been associated with BEEC, including terminal deletions of 1q, 1p36, and 9q34.1. The strongest associated chromosomal abnormality involves 22q11.21 microduplications, accounting for ~3% of BEEC cases [67,72]. Genome-wide association studies have documented genes significantly associated with BEEC, including WNT3, WNT9B, and TP63, which regulate urorectal and urogenital development [67,73,74,75]. Mutations in the ISL1 gene were also significantly associated with bladder exstrophy in multiple meta-analyses, and ISL1 appears to impact genital tubercle development through downstream effects on Fgf10, Wnt5a, and Bmp4 [67,76,77,78].

#### 3.2.3. Obstructive Uropathy (Posterior Urethral Valves, Ureteropelvic Junction Obstruction)

##### Epidemiology

Obstructive uropathy is a rare condition that prevents normal flow through the urinary tract. Lower urinary tract obstruction (LUTO) has an estimated incidence of 2–3 per 10,000 births, with the most common subtype being posterior urethral valves (PUV) [5,79,80]. Ureteropelvic junction obstruction (UPJO) has an incidence of 0.5–1 per 1000 and is associated with multiple other urinary tract anomalies [52,81].

##### Clinical Presentation

While the point of obstruction varies between different types of obstructive uropathy, a common characteristic is dilation of the renal pelvis due to decreased outflow from the kidneys. LUTO often presents with the triad of megacystitis, a dilated posterior urethra, and hydronephrosis. However, UPJO generally presents with only hydronephrosis due to the proximal location of the obstruction [81,82]. Additional presentations include ureterovesical junction obstruction (UVJO), defined by an obstruction in the distal 1–2 cm of the ureter [83], and primary non-refluxing megaureter (also known as idiopathic megaureter), which presents with isolated dilation of the ureter [84,85].

Prognosis can be challenging to define explicitly; however, outcomes are worse if oligohydramnios is present before 20 weeks gestation due to impaired fetal lung development. Perinatal complications remain a significant issue, with mortality rates as high as 80% if untreated [79].

##### Pathophysiology

PUVs occur when membranous folds attached to the posterior urethral wall obstruct the exit to the urethra, leading to dilation of the bladder and more proximal structures [86]. Type I PUVs are the most common variation, arising from remnants of the Wolffian duct that fuse anteriorly. Type III PUVs are caused by a membrane at the caudal verumontanum with a central hole either distal or proximal to the verumontanum. Hypertrophic plicae colliculi present as bicuspid leaflets of the verumontanum and were historically described as type II PUV, although they are no longer classified as true PUV. Cobb’s Collar is another potential classification that is not a true valve but instead a congenital bulbar urethral stricture [87].

Genes that have been suggested as potentially playing a role in PUV include PCDH9, SALL1, BNC2, TBX5, and PTK7 [88,89,90]. The PCDH9 gene is responsible for cell adhesion in neural tissue; however, it is also expressed in the developing urinary tract [88]. SALL1 is involved in multiple forms of CAKUT and Townes–Brocks syndrome, as described previously [36,88]. Genome-wide analysis has suggested the involvement of the TBX5 and PTK7 genes [90]. There is also a report of partial chromosome 11 duplication, possibly leading to PUV development [91].

Multiple pathologic processes can cause UPJO during development, but the most common is aperistaltic ureteral segments, which have also been suggested to be involved in the pathophysiology of idiopathic megaureter. This is theorized to be caused by hypertrophy or the absence of ureteral smooth muscle. Other causes include acquired obstruction with stricture/fibrosis or crossing vasculature interfering with or compressing the ureter, although the latter explanation is controversial [81,82,85,92,93]. In partial UPJO, cytokines and vasoactive peptides such as IL-5 and eotaxin-2 lead to increased inflammation and a subsequent decrease in total GFR [82]. As renal tubular dilatation leads to inflammation followed by injury and fibrosis, renal tubular apoptosis may occur through activation of TGF-β1, TNF-α, FAS, p54, caspases, and ceramide. Removal of obstruction via surgical intervention showed some improvement in renal function, but not total recovery [94].

#### 3.2.4. Ureteroceles

##### Epidemiology

Ureteroceles are defined as congenital cystic dilatations of the intravesical ureter within the bladder and occur four to six times more frequently in women. The overall incidence of these conditions is estimated to be 1 in 500 to 4000. The majority of ureteroceles are unilateral and intravesicular; however, a minority are ectopic with insertion points other than the bladder. Additionally, most cases are observed with duplicated collecting systems [95,96].

##### Clinical Presentation

Intravesical ureteroceles are either stenotic or nonobstructed. A subset of stenotic ureteroceles can present with obstruction and a small ureteric orifice, whereas nonobstructing ureteroceles display a large open orifice [95,96]. Ectopic ureteroceles are more variable in presentation. Sphincteric ureteroceles are most often inserted in the bladder neck proximal to the external sphincter. A sphincterostenotic presentation is similar; however, obstruction is observed. Cecoureteroceles insert within the bladder, but the dilated distal ureter travels through the bladder to obstruct the bladder neck. Blind ectopic ureteroceles extend into the bladder neck, often leading to obstruction of the bladder and direct drainage from the kidney [95,96]. Symptoms of ureteroceles can be difficult to differentiate from other processes, but failure to thrive, UTIs, abdominal mass due to bladder distension, and urosepsis should prompt consideration of the diagnosis. A detailed family history is crucial, as it may increase suspicion. Occasionally in females, a ureterocele may prolapse and present as a vaginal mass.

##### Pathophysiology

During normal development, the ureteral bud forms from the mesonephric duct and subsequently develops into the renal collecting system. The distal ureter is occluded by a membrane that dissolves during normal development, but it is theorized that this membrane fails to dissipate in ureteroceles. However, numerous variations observed in ureterocele presentation suggest that the underlying process may be more complex [64,95,97]. Ectopic ureteroceles present in a wide range of locations, but they most frequently follow along the migratory path of the mesonephric duct. Generally, ectopic ureteroceles are considered sporadic without genetic etiology; however, cases have been associated with Zinner’s syndrome [98,99].

In the event that two ureteral buds develop, a duplicate collection system forms. The Weigert–Meyer law describes the flow of urine from the kidneys into the bladder in the setting of dual collection systems, which are frequently associated with ureteroceles. Ureters from the cranial moiety drain into distal and medial orifices, and the caudal moiety drains into proximal and lateral ureters. The ureter draining the cranial moiety is more likely to develop ureterocele, whereas the caudal ureter is more likely to present with VUR [96,97].

Ureteroceles have been observed to have familial presentations, suggesting there may be a genetic component to this condition [64,100]. Gene mutations associated with the development of ureteroceles include GDNF and c-ret, both of which are implicated in other forms of CAKUT. Increased GDNF expression is associated with the formation of two or more ureteric buds, which may be impacted by activation of the Eya1, Pax2, and Sall1 transcriptional factors. The FoxC1 and FoxC2 transcription factors have also been implicated in the formation of multiple ureteric buds if signaling is halted by the Slit 2 protein, with a resultant increased risk of ureteroceles. As discussed previously, the agtr2 gene is associated with the formation of duplex collecting systems and, therefore, also conveys an increased risk of ureteroceles [64].

## 4. Conclusions

CAKUT represents a broad range of disorders with significant clinical impact on patients. Advancements in our understanding of genetics have provided insight into the pathophysiology of these processes. A multidisciplinary approach involving basic sciences, genetics, clinical, pharmacologic, and statistical research has progressed our knowledge significantly. With sophisticated therapeutic target identification and genetic editing technology becoming more prevalent, a stronger understanding of genetic mechanisms will guide future studies.

## Figures and Tables

**Table 1:** CAKUT classification: nephropathies and uropathies.

Nephropathies	Uropathies
Unilateral renal agenesis	Multiple ureters
Bilateral renal agenesis	Epispadias
Multicystic dysplastic kidneys (MCDK)	Bladder exstrophy
Simple renal hypoplasia	Cloacal exstrophy
Oligomeganephronia	Posterior urethral valves (PUV)
Horseshoe kidney	Ureteropelvic junction obstruction (UPJO)
Ectopic kidney	Ureterocele
Malrotated kidney	

**Table 2:** CAKUT diseases, epidemiology, mutations, mechanisms, and outcomes.

Disease	Genetic Mutations	Mechanism	Symptoms/ Outcome	Associated Syndromes/Notes
Unilateral renal agenesis	CFTR *, ADGRG2 *, WT1 *, RET ^, CHD1L *, TRIM32 *, WNT, WNT4 ^, PAX8 *	Failure of the ureteric bud to induce metanephric mesenchyme	Frequently asymptomatic. Less often hypertension, compensatory hypertrophy of solitary kidney, CKD, UTI	Zinner syndrome, OHVIRA, VACTERL
Bilateral renal agenesis	RET^, GDNF, WNT11, DSTYK, ITGA8, FGF20, GREB1L, WT1, ANOS1 *, EYA1 *, SIX1 *, SIX5 *	Developmental failure of both ureteric buds	Rapidly fatal, oligohydramnios, pulmonary hypoplasia, Potter phenotype	
Multicystic dysplastic kidneys	PAX2, TCF2, calcineurin-NFAT, BMPER	Abnormal interaction between the ureteric bud and the metanephric mesenchyme	VUR, UTI, Potter phenotype, hypertension, progression to renal failure	VACTERL, renal coloboma, branchio-oto-renal syndrome, Mayer-Rokitansky
Simple renal hypoplasia	BMP2, BMP4, ALK3	An underdevelopment of renal tissue due to insufficient nephron formation in utero	Frequently asymptomatic. VUR, hypertension	
Oligomeganephronia	PAX2 *, EAY1 *, SALL1 *, RET1, chromosome 4 *	Unknown, but hypothesized as reduced nephron number during renal development	Short stature, polyuria, polydipsia, proteinuria, progressive CKD, hypertension, glomerular hypertrophy	Renal-coloboma syndrome, branchio-oto-renal syndrome, Townes-Brocks syndrome, acrorenal syndrome, and Seckel syndrome,
Horseshoe kidney	No known genetic etiology	Fusion of inferior poles of kidney	Frequently asymptomatic. VUR, nephrolithiasis, UTI, hydronephrosis	Turner Syndrome, Trisomy 18
Ectopic kidney	No known genetic etiology	Abnormal migration of the kidney during development	Frequently asymptomatic. VUR, nephrolithiasis, urinary incontinence, UTI, hydronephrosis.	Omphalocele–Exstrophy-Imperforate Anus–Spinal Defects Syndrome (OEIS), MCDK, Ureterocele
Multiple ureters	RET, GDNF, GATA3, SLIT2/ROBO2, FOXC1, FOXC2, SOX11, GREM1, BMP4, beta-catenin	Premature bifurcation of the ureteric bud or two distinct ureteral buds.	VUR, UTI, hydronephrosis, ureteroceles, nephrolithiasis	Associated with ureteroceles
Epispadias	WNT3, WNT9B, TP63, ISL1	Failure of midline fusion of the genetic tubercle	Urinary incontinence, cosmetic concerns, sexual dysfunction	Frequently presents with bladder exstrophy
Bladder exstrophy	WNT3, WNT9B, TP63, ISL1	Improper closure of the mesoderm development between bladder and abdominal wall	Urinary incontinence, hydronephrosis, UTI, sexual dysfunction	Epispadias, pelvic floor defects
Cloacal exstrophy	WNT3, WNT9B, TP63, ISL1	Severe disruption in the closure of the ventral abdominal wall and cloacal membrane	Urinary + bowel incontinence, obstructive uropathy, renal failure, fistula formation	Spinal anomalies, genital malformation
Posterior urethral valve	PCDH9, SALL1, BNC2, TBX5, PTK7	Formed by remnants of the Wolffian duct or failure of the urogenital membrane to dissolve	Oligohydramnios, hydronephrosis, Potter phenotype, UTI, CKD	Exclusive to males
Ureteropelvic junction obstruction	No known genetic etiology	Aperistalsis of ureteral segments due to hypertrophy or absence of ureteral smooth muscles	Oligohydramnios, hydronephrosis, Potter phenotype, renal failure	
Ureteroceles	RET/GDNF, EYA1, PAX2, SALL1, FOXC1, FOXC2, SLIT2/ROBO2, AGTR1	Failure of membrane at the distal ureter to completely dissolve and its subsequent dilation	Asymptomatic, bladder distension, urosepsis	Dual renal collecting systems

* Associated with a specific congenital syndrome; ^ primary gene disturbance.

## Data Availability

The data presented in this study are available from the PubMed and th National Library of medicine at https://pubmed.ncbi.nlm.nih.gov/, accessed on 29 September 2024.
